# *Salmonella enterica* Serovar Enteritidis Control in Poultry Litter Mediated by Lytic Bacteriophage Isolated from Swine Manure

**DOI:** 10.3390/ijerph18168862

**Published:** 2021-08-23

**Authors:** Paula Rogovski, Raphael da Silva, Rafael Dorighello Cadamuro, Estêvão Brasiliense de Souza, Beatriz Pereira Savi, Aline Viancelli, William Michelon, Deisi Cristina Tápparo, Helen Treichel, David Rodríguez-Lazaro, Gislaine Fongaro

**Affiliations:** 1 Laboratory of Applied Virology, Department of Microbiology, Immunology and Parasitology, Federal University of Santa Catarina, Florianópolis 88040-900, SC, Brazil; paularogovski@gmail.com (P.R.); rapha.silva1596@gmail.com (R.d.S.); rafaelcada@hotmail.com (R.D.C.); estevaobrasiliense@gmail.com (E.B.d.S.); beasavis2@gmail.com (B.P.S.); 2 Research Group on Engineering, Performance and Environmental Quality, Universidade do Contestado (PMPECSA), Concórdia 89711-330, SC, Brazil; alinevbortoli@gmail.com (A.V.); william@unc.br (W.M.); 3 Centro de Engenharias e Ciências Exatas, Western Paraná State University, Cascavel 85819-110, PR, Brazil; deisictapparo@gmail.com; 4 Laboratory of Microbiology and Bioprocesses, Federal University of Fronteira Sul, Erechim 89802-112, RS, Brazil; helentreichel@gmail.com; 5 Division of Microbiology, Department of Biotechnology and Food Science, University of Burgos, 09001 Burgos, Spain; 6 Research Centre for Emerging Pathogens and Global Health, University of Burgos, 09001 Burgos, Spain

**Keywords:** environmental management, food safety, bacteriophage isolation, *Salmonella*, foodborne pathogen control

## Abstract

We report the use of bacteriophages for control of *Salmonella* Enteritidis in poultry production. Phage was isolated by the double-agar plate assay from agricultural waste samples, and one isolate, named SM1, was selected and propagated for application in poultry litter. Two experimental protocols were tested: single treatment and repeated treatment (re-application of phage SM1 after 6 h and 12 h). Each treatment cycle involved 25 g of poultry litter placed in plastic boxes and contaminated with 10^5^ Colony Forming Units mL^−1^ (CFU mL^−1^) of *S.* Enteritidis, in independent duplicates. The contaminated litter was treated with 10^6^ Plaque Forming Units mL^−1^ (PFU mL^−1^) of SM1 phage by dripping. Repeated application of phage SM1 reduced *Salmonella* counts by over 99.9%; the phage persisted in poultry litter for over 35 days. This study illustrates the application of SM1 treatment as a promising technology for bacterial control in production matrices that could allow safe and sustainable use of agricultural waste products as biofertilizers.

## 1. Introduction

The sustained acceleration of population growth worldwide, modern lifestyles, and eating habits have intensified human contact with animals and animal food-derived products, increasing the risk of the human population being infected by foodborne pathogens [1,2]. More than 70% of emerging and re-emerging diseases have zoonotic origin, and these diseases cause about a million deaths per year and about a billion cases of human and animal illness worldwide [3,4,5]. Some of the feeding and confinement methods used, and the lack of disease-control protocols, contribute to the persistence and spread of zoonosis [6]. An aggravating factor is the increasing prevalence of bacterial antibiotic resistance, now a global concern for human and animal health [7]. The inappropriate and extensive use of antimicrobials in agriculture, associated with the livestock environment, promote the spread of multi-drug resistant bacteria [8,9].

Various species of Enterobacteriaceae family are major foodborne pathogens. They are resilient and can survive in diverse conditions, e.g., a wide range of pH and temperature, and consequently are present in numerous ecological niches [10,11]. The Enterobacteriaceae group includes over 50 genera and 210 species, some of which are of particular public health concern as they are the main zoonotic pathogens associated with chronic and acute gastrointestinal disease; the *Salmonella* genus causes a large number of infections, for example 46,623 reported cases of salmonellosis in 2016 in the USA, and 87,923 cases in 2019 in Europe [12,13]. *Salmonella* spp. are also threatening global animal production [13]. Animals can be infected by bacteria from various sources, including insect vectors, contaminated food, or during traveling and trade; early detection could reduce economic losses, especially in asymptomatic cases [14]. 

Several different approaches to preventing bacterial contamination have been proposed, aiming to reduce environmental contaminations and promote animal health. Since the discovery of viruses that are able to infect bacteria, known as bacteriophages or phages, they have been applied successfully for various bacterial control purposes [15,16].

One of these applications is the use of phage for food sanitization. Indeed, phage is being increasingly used in the food production chain for food products, such as meat and vegetables [17,18,19,20]. Phage is potentially of value for environmental purposes, for both bacterial control and as indicators of processing efficiency [21,22]. Phages have various properties favorable for their application in the environment: they have self-regulating activity, low toxicity to eukaryotic cells, and cannot regenerate the host target due to bacterial lysis [23,24,25]. The One Health approach emphasizes the connection between animal, environmental, and human health, aiming to reduce the problems that affect the system at all levels [26]. The One Health strategy considers the multifactorial and multisectoral aspects of the spread of antimicrobial resistance and promotes control through different actions. The research for alternative tools to decrease the use of antibiotic drugs is therefore important [27]. Here, we report a study of the stability and control of *Salmonella enterica* serovar Enteritidis in poultry litter by application of a lytic bacteriophage isolated from swine manure.

## 2. Materials and Methods

### 2.1. Environmental Sampling and Bacteriophage Isolation

Five liters of swine manure were collected from livestock farms in the West Region of Santa Catarina, Brazil, one of the major meat production zones in Brazil. Manure samples were stored at 4 °C until bacteriophage isolation. Bacteriophages were isolated from samples according to ISO 10705-1:1995 [28], using *Salmonella enterica* serovar Typhimurium (ATCC^®^ 14028™) as host. Briefly, swine manure samples were diluted in PBS (phosphate buffer, pH 7.0), and then filtered through 0.22 μm cellulose filters; 1 mL of the filtrate was mixed with 1 mL of log-phase *S.* Typhimurium culture (Optical Density 0.6) and the samples were each added to 1 mL of solid BHI-agar (1.2% liquid at 50 °C). The solution was incubated at 37 °C for 12–16 h to allow the appearance of plaques.

#### 2.1.1. Bacteriophage Propagation and Selection

The plate containing different phage plaque profiles was picked, segregated in sterile tube and mixed with 5 mL of SM buffer (50 mM Tris HCl, pH 7.4; 0.1 M NaCl; 8 mM MgSO_4_; pH 7.5), incubated on a rocker at 4 °C for 12 h, and then eluted by centrifugation (10,000× *g* for 5 min at 4 °C). Then, 1 mL of the filtered eluate was added to 25 mL of LB (Luria Bertani) medium with 1 mL of *S. e.* Typhimurium (0.6 OD) as the host. The infection was performed during 18h for bacteriophage propagation. After three rounds of propagation, the phage titer was determined and expressed in plaque forming units (PFU). After this, the isolated bacteriophages were evaluated as the lyse capacity and the natural titer increasing capacity until 18 h. Among the five different PFU profiles, according to size and shape, one isolated denominated phage SM1 was quantified (1.8 × 10^7^ PFU mL^−1^), propagated and used for bacterial control experiments being applied in contaminated poultry litter.

#### 2.1.2. Molecular Identification of the Bacteriophages

For the molecular identification of phage SM1, the culture medium solution (LB) containing the phage was filtered through a cellulose filter (0.22 μm) and then the viral nucleic acid was extracted with the commercial kit PureLink Viral RNA/DNA (Invitrogen-Life Technologies). Then the extracted genetic material was sent to Neoprospecta Inc., Florianópolis, Brazil, for the sequencing via MiSeq—Illumina, and analysis of the generated data. The assembly of genomes and searching in databases was performed by trimming the raw data with Prinseq and the search for families and viral species with specificity for bacteria was done using the Kaiju taxonomic classification program, with RefSeq as the sequence bank.

### 2.2. Poultry Litter and Eggshell Additive

Poultry litter were collected in six different poultry farms distributed in the West Region of Santa Catarina, Brazil. The poultry litter samples were homogenized, and random samplings were performed in duplicates for their use in the treatment tests. A second collection was carried out to obtain built up poultry litter, used during different broiler flocks (one flock correspond to a poultry production cycle, that lasts about 40–45 days, with addition of 5% of limestone at its end). Poultry litter from 1, 3, 6, and 9 flocks were used to test SM1 efficiency against *S. e.* Enteritidis, as well to test the SM1 stability in function of time.

Eggshells residues were used as alternative carbon source additive to poultry litter from 9 flocks. For this, eggshells were collected from agriculture chain production, dried at 280 °C and mills in sizes of 300 μM, being then added at 10% *v*/*v* at poultry litter for tests.

Poultry litter were known negative for *Salmonella* spp., and were artificially contaminated with *S. e.* Enteritidis wild type (isolated from contaminated poultry litter samples as ISO 6579-1: 2017, identified using the commercial Painel G (Porobac) and propagated in LB medium) [29]. All poultry litter were evaluated according to its physicochemical characteristics, considering total, fixed, and volatile solids content, pH, and ammoniacal nitrogen (NH_3_-N), according to APHA, 2012 [30].

### 2.3. Stability of Phage SM1 in Poultry Litter

The stability of phage SM1 was evaluated on built up poultry litter, considering litter of 1, 3, 6, and 9 flocks. In addition, poultry litter from 9 flocks was added with 10% *v*/*v* eggshells for carbonating purpose. For this, 100 ± 0.05 g (Gehaka AG-200 Analytical balance-10 mg—199.999 g) of each poultry litter samples were placed in sterile plastic recipients (forming a 1 cm layer), in duplicates. All poultry litter were tested for the assurance of no *Salmonella* spp. Then, the litter were inoculated with 10 mL of the solution containing 3 × 10^7^ PFU mL^−1^ of SM1. The stability of the phage was assessed by means of the PFU counting, using the double-agar plate test according to ISO 10705-1: 1995, for 35 days [29].

### 2.4. S. e. Enteritidis Inactivation in Poultry Litter Using Phage SM1

On the *S. e.* Enteritidis inactivation test, for each treatment, 25 g ± 0.05 g of poultry litter (free of *Salmonella* spp.) were weighed in a Gehaka AG-200 Analytical balance-10 mg–199.999 g. The poultry litter was placed in boxes to form a 1 cm layer, artificially contaminated by dripping with 2,5 mL of *S. e.* Enteritidis solution, containing 1 × 10^6^ colonies forming units (CFU mL^−1^), in duplicates. After 1 h at 25 °C for acclimation, the poultry litter were treated by dripping 10 mL of the phage SM1 solution, with the viral title of 1.8 × 10⁶ PFU mL^−1^. The controls of the experiment were poultry litter without *S. e.* Enteritidis inoculation (phage control) and poultry litters with *S. e.* Enteritidis inoculation without phage SM1 application (bacterial control).

**Figure 1:**
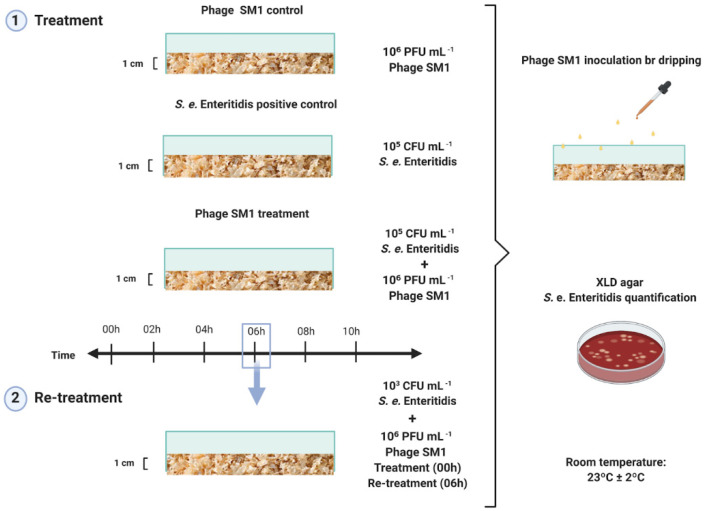
Scheme of the experimental system, with the treatments and controls.

For *S.* *e*. Enteritidis and SM1 phage enumeration from poultry litter, 1 ± 0.01 g of samples (weighed on a Shimadzu AUW220D-1 mg–220 g analytical balance) was randomly collected with a sterile spatula to be evaluated as a function of treatment time of poultry litter, considering samples treated with Phage SM1 and control of the tests). *S. e.* Enteritidis concentration was determined using the XLD agar (Xylose, Lysine Deoxycholate agar—Kasvi), as selective and differential medium on 1:10 and 1:100 dilutions, after 24 h and 48 h of incubation in a bacteriological incubator at 37 °C, according to ISO 6579-1: 2017 [29]. The phage SM1 in experiment were enumerated by the double-layer agar overlay method, in function of the time, using *S. e.* Typhimurium as the host. The PFU plates were incubated in a bacteriological incubator at 37 °C until 6 h, and the PFU were enumerated (Figure 1).

### 2.5. Phage SM1 Re-Treatment of Poultry Little

A re-treatment assay was performed using low bacterial concentration (3.0 × 10^3^ CFU/mL^−1^) due to the resurgence of the bacterial colonies after the first phage SM1 treatment. For this purpose, a second dose of phage SM1 was added 6h after the first SM1 application by dripping (10 mL of the Phage SM1 solution, 1.8 × 10⁶ PFU mL^−1^), as shown in Figure 1. The bacteriophage and bacterial quantification along the time were performed as described previously on item 2.5.

### 2.6. Statistical Analysis

A linear regression test was applied to evaluate the behavior of phage SM1 and S. e. Enteritidis reduction on poultry litter in function of the time (h). ANOVA tests were performed to compare the groups—treated with phage and untreated. The T test was used for physicochemical parameters analyses between the different flocks, treated with phage and untreated. All tests were performed in the software Prism 6 (GraphPad, San Diego, CA, USA), and significant differences were considered when *p* ≥ 0.05.

## 3. Results and Discussion

### 3.1. Bacteriophage Isolation, Propagation and Selection

The isolation process resulted in five distinguished plaque profiles, amongst which after three replication rounds, one was selected. This phage was denominated SM1 due to its isolation from a swine manure sample. It is important to consider that swine manure possesses a rich microbial composition. This approach demonstrates the integrated agriculture concept, using organisms and/or molecules from the husbandry environment as a way of waste valorization within the production chain. Phage SM1 titter was quantified in 1.8 × 10^7^ PFU mL^−1^ and aliquots were prepared for application and storage on −80 °C freezer.

### 3.2. Molecular Identification of Bacteriophage 

The molecular identification of the phage SM1 showed its taxonomic classification as part of the *Caudovirales* order, *Siphoviridae* family (GenBank number 2478666). The genetic analyses enabled the phage SM1 host range identification, endorsing its classification as a Salmonella virus, or F-specific phage. In addition, any integrase sequences were found, reassuring the phage SM1 lytic profile.

### 3.3. Stability of Phage SM1 in Poultry Litter

**Figure 2:**
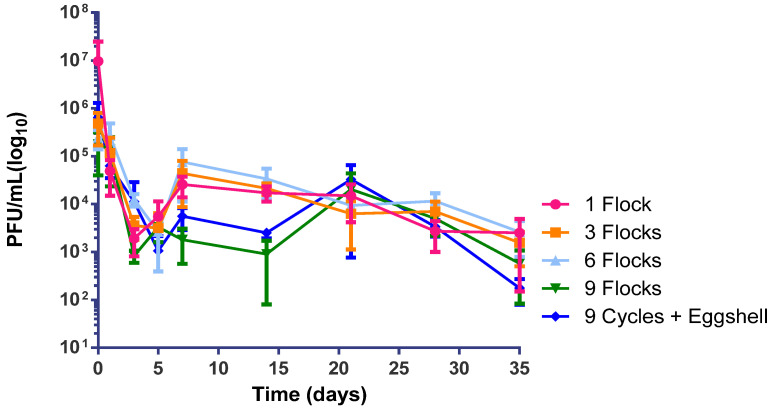
Phage titer over the time after application of phage SM1 in poultry litter with different built-up levels and the addition of an alternative carbonate (eggshell).

In order to investigate the long-term permanence of phage SM1 in poultry litter, phage titration was monitored for a period of 35 days, considering built up poultry litter from 1, 3, 6, and 9 flocks of poultry production, and thus poultry litter from 9 flocks added with eggshell as alternative alkalinizing treatment (Figure 2). The SM1 in poultry litter from 9 flocks added with 10% of eggshell showed a subtle decay after 20 days of the test. However, there were no significant differences of SM1 stability among the poultry litter flocks treatments (*p* > 0.05).

In addition, the physicochemical characterization of the poultry litter after the phage SM1 application is presented in Table 1. There were no significant differences among treatments (*p* > 0.05).

### 3.4. S. Enteritidis Inactivation in Poultry Litter Using Phage SM1

**Figure 3:**
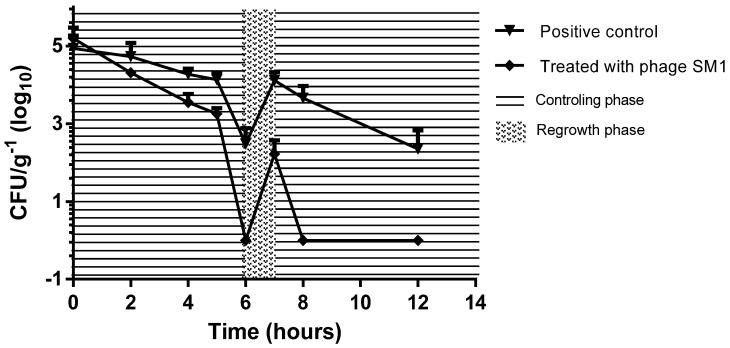
*S. e.* Enteritidis colonies counting after phage SM1 application on poultry litter. The three-phase behavior of the bacterial count after a single phage use shows the *S. e.* Enteritidis regrowth potential.

The poultry litter used was tested before the addition of phage SM1 and did not contain bacteriophages capable of infecting *S. enterica* serovar Enteritidis. Thus, the bactericidal effects observed were due to phage SM1. Numerous studies have already shown that phages can be used effectively for *Salmonella* spp. control in poultry, although in most of these studies, administration is through drinking water; and some use phage directly for meat sanitization [18,19,20]. Other phage applications for *Salmonella* spp. control in foods, such as vegetables and juices, indicated the potential of phages as options to reduce the use of chemical additives in the control of food bacteria [17,19,31]. Our study demonstrates that SM1 phage treatment is effective against *S. enterica* in contaminated poultry litter. S. Enteritidis counts fell by 3log_10_ following phage SM1 applications. However, we followed S. Enteritidis in the treated poultry litter for 12 h—three-phase of bacteria behavior was observed, one with significant reduction of bacteria (3 ± 0.3 log_10_ reduction), between 2 h and 6 h after treatment (*p* = 0.0016), one of regrowth phase (1.5 ± 0.2 log_10_ increase) between 6–7 h after the phage application and, one new bacterial control (3 ± 0.3 log_10_ reduction) between 8–12 h after treatment. All experiments were run with untreated samples (without phage SM1 inoculation) as controls (Figure 3).

Bacterial regrowth is a problem even for antibiotic treatments and may require subsequent doses. Populations of some bacterial species, including *S. enterica* serovar Enteritidis, present persistent cells, which may be a cause of antibiotic failure. Indeed, repeated antibiotic administration favors selection of resistance, and this may result in lack of success of the drug treatment [32,33,34]. One of the determining factors for interaction between phage and bacteria is bacterial motility, so infection of the bacteria by phage may be hampered in drier conditions such as those in poultry litter (45 ± 10%) [35,36,37].

### 3.5. Phage SM1 Re-Treatment of Poultry Litter

**Figure 4:**
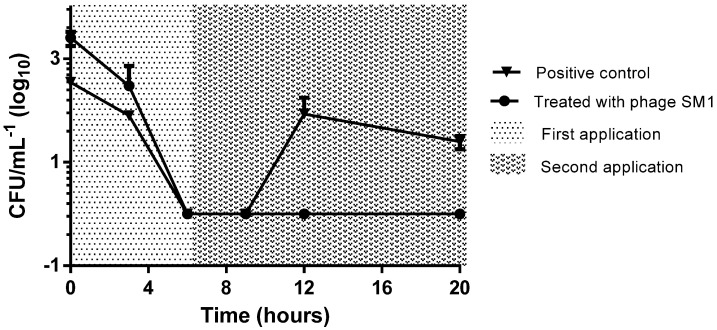
Efficacy of two sequent applications of phage SM1 for *S. e.* Enteritidis reduction on poultry litter. The positive *S. e.* Enteritidis control is presented by triangles, indicating the *S. e.* Enteritidis colonies enumeration on poultry litter without phage SM1 addition.

Due to the observation of the *S. e.* Enteritidis colonies regrowth, a new experimental set was performed, with the reapplication of the initial dose (1.8 × 10^6^ PFU mL^−1^) of the phage SM1, 6 h after its first application. In this experiment, the untreated control showed the same *S. e.* Enteritidis regrowth behavior, with a reduction of 3 ± 0.1 log_10_ followed by an increase of 2 ± 0.3 log_10_ in the bacterial count. However, the second application of the phage SM1 was able to avoid the resurgence of *S. e.* Enteritidis colonies, which reached an undetectable value (*p* = 0.0043). Thus, the reapplication of the phage was able to maintain the reduction of 3log_10_ reached 6 h after the first contact with the poultry litter (Figure 4).

The *Salmonella* population dynamics described above show the importance of high bacteriophage titers for effectiveness. The multiplicity of infection used in this study was 10 phage particles for each bacterial cell (MOI 10:1). A single phage application did not prevent regrowth, although re-application 6h after the first treatment did (3log_10_ reduction). Similarly, Ahmadi et al. reported that administration of bacteriophages (10^9^ PFU/mL^−1^) on 3 consecutive days were able to eliminate *S.* Enteritidis [37]. The protective effects of phage against *S.* Enteritidis and *S.* Typhimurium have also been reported in analyses of their combined action with probiotics or when added to poultry food [38,39,40]. On the other hand, the environmental survival of the phage must be accessed because it depends on the nature of the virus itself, being influenced by the surrounding conditions, such as the pH, sunlight, ionic strength, or temperature [41].

Phages, such as phage SM1, could be useful for maintaining a healthier environment for livestock, because used poultry litter tends to be contaminated with *Salmonella* spp., and these pathogens can persist 6 months or more in soils amended with this residue [42,43]. Such persistent bacterial contaminations hamper implementation of integrated agriculture, as reflected by the One Health Approach: clearly, it is unsafe to grow food in contaminated environments. Fortunately, the advent of phage-based techniques is promising to combat antimicrobial resistance, and could also contribute to the safety of using poultry litter and other agricultural waste products. This use would be beneficial for nutrient recovery and could consequently have economic benefits in the production chain, reducing the use of chemical fertilizers, while decreasing the use of antimicrobial drugs in animal husbandry.

## 4. Conclusions

We report the application of SM1 phage to poultry litter to reduce the prevalence of *Salmonella* in poultry production environments (poultry litter). Our study contributes to the progress towards the use of bacteriophage in the agro-food sector: in particular it may be possible to convert agricultural animal waste, in this case poultry litter, into a useful and safe product (fertilizers), and thereby improve recycling of nutrients in the context of a circular economy.

## Figures and Tables

**Table 1:** Physico-chemical characterization of poultry litter before and 35 days after phage SM1 application.

Samples	pH	NH_3_—N (mg L^−1^)	TS (g kg^−1^)	VS (g kg^−1^)	N (mg kg^−1^)	P (mg kg^−1^)	K (mg kg^−1^)
1 Flock poultry litter	8.16 ± 0.06	1687 ± 8	892.08 ± 1.46	748.51 ± 3.01	34,450 ± 228	21,316 ± 220	10,183 ± 78
3 Flocks poultry litter	7.73 ± 0.04	1711 ± 45	890.72 ± 23.55	691.04 ± 19.18	36,054 ± 140	22,601 ± 230	11,672 ±57
6 Flocks poultry litter	8.43 ± 0.04	2091 ± 20	866.69 ± 10.89	678.53 ± 35.83	35,963 ± 178	23,670 ±195	11,757 ± 38
9 Flocks poultry litter	8.61 ± 0.13	3545 ± 158	880.02 ± 0.14	652.48 ± 2.65	28,883 ± 120	27,992 ± 165	14,279 ±46
1 Flock poultry litter + SM1	8.38 ± 0.11	2468 ± 18	892.17 ± 1.95	626.13 ± 13.80	32,906 ± 200	23,700 ± 209	12,026 ±29
3 Flocks poultry litter + SM1	7.72± 0.01	2274 ± 65	884.57 ± 6.44	719.68 ± 15.40	38,383 ± 190	21,641 ± 187	12,060 ± 69
6 Flocks poultry litter + SM1	7.79 ± 0.07	1887 ± 124	895.16 ± 1.33	751.26 ± 4.64	35,471 ± 179	18,878 ± 267	10,718 ± 58
9 Flocks poultry litter + SM1	8.88± 0.01	3838 ± 165	885.71 ± 0.58	636.95 ± 16.03	24,096 ± 250	25,349 ± 278	15,127 ± 110
